# Random Deep Belief Networks for Recognizing Emotions from Speech Signals

**DOI:** 10.1155/2017/1945630

**Published:** 2017-03-05

**Authors:** Guihua Wen, Huihui Li, Jubing Huang, Danyang Li, Eryang Xun

**Affiliations:** School of Computer Science and Engineering, South China University of Technology, Guangzhou, China

## Abstract

Now the human emotions can be recognized from speech signals using machine learning methods; however, they are challenged by the lower recognition accuracies in real applications due to lack of the rich representation ability. Deep belief networks (DBN) can automatically discover the multiple levels of representations in speech signals. To make full of its advantages, this paper presents an ensemble of random deep belief networks (RDBN) method for speech emotion recognition. It firstly extracts the low level features of the input speech signal and then applies them to construct lots of random subspaces. Each random subspace is then provided for DBN to yield the higher level features as the input of the classifier to output an emotion label. All outputted emotion labels are then fused through the majority voting to decide the final emotion label for the input speech signal. The conducted experimental results on benchmark speech emotion databases show that RDBN has better accuracy than the compared methods for speech emotion recognition.

## 1. Introduction

Emotions accompany human being in the life everywhere and every moment [1]. They can be recognized and communicated through speech signals that constitute 38% of the whole communicated emotions [2]. This is why speech emotion recognition (SER) has been recently emphasized that automatically classifies the emotional state of a speaker from speech signals into one of several basic emotions [3, 4]. SER has been applied to deal with the issues in many fields. For example, it can be applied to design a medical robot that provides the better health-care services for patients by continuously monitoring the patients' emotional state [5] and provides diagnostic suggestions for therapists [6]. SER can be implemented through machine learning methods that is composed of both speech feature extraction and classification. The speech feature extraction is a key issue for all classification methods to obtain better generalization [7]. The extracted features should minimize the distances between samples with the same emotion class and maximize the distances between samples with the different emotion classes [8]. If the features are not well defined, the best classifier could have difficulty in reaching the good performance. Most typical features are predefined by hand-engineered ones, including newly proposed nonlinear dynamic features [3]. They have achieved the great success in specific fields where the small speech training data can be available only. However, these features perform inconsistently on different emotion recognition tasks [9]. They are in lower level so as to make themselves difficult to extract and organize the discriminative features from the speech signals. As a matter of fact, it is not clear which speech features are most powerful in distinguishing emotions [2, 9]. They are easily influenced by speakers, speaking styles, sentences, and speaking rates, because these factors directly affect the extracted speech features such as pitch and energy contours [5]. Besides, they are not easily tuned for the newly coming speech signals. Speech emotions tend to have overlapping features, making it difficult to find the correct classification boundaries. To deal with these issues, deep learning methods can be considered that can automatically discover the multiple levels of representations in speech signals. For example, it utilizes the higher level features to represent the more abstract concepts [10]. This is the reason that they succeed in breaking most of the world records of the recognition tasks. Among deep learning methods, deep belief network (DBN) is the most representative one [11, 12]. It applies the unsupervised learning algorithms such as auto-encoders and sparse coding to learn higher level feature representations from the unlabeled data [13]. It has produced the state-of-the-art results on recognition and classification tasks [10]. On the other hand, typical classification methods used for speech emotion recognition include hidden Markov model (HMM) [14], Gaussian Mixture Model (GMM) [15], artificial neural networks such as recurrent neural network (RNN) [16], support vector machine (SVM) [17, 18], and the fuzzy cognitive map network [19]. These methods are confronted with the complicated decision boundary of the classification. In such case, the ensemble learning can be applied that can learn any nonlinear boundary through appropriately combining the simple classifiers. It has potential ability to greatly reduce overfitting problems, to decrease the risk of a single classifier, and to obtain better performance than its single classifiers [20]. The usual ensemble classifiers are boost-based, bagging-based approaches [21], random subspace [22], and so forth. Some of them have been applied to perform speech emotion recognition but still fail to reach the performance as expected. For example, it seems that random forest and AdaboostDT have the bad effect for speech emotion classification [23]. The possible reason is that the diversity of the base classifiers is not guaranteed [24]. As to random subspace, the classifiers trained with different features should have certain diversity inherently. However, this assumption is not always true. For instance, there are two different features sets, but the classifiers trained by the two features sets may have the similar classification results, leading to no rich diversity between them [24]. To ensure the diversity among base classifiers, the features in random subspace should be further abstracted from different viewpoints using DBN. Therefore, this paper presents a novel random deep belief network (RDBN) method for speech emotion recognition, which is composed of the random subspace, DBN, and SVM within the framework of ensemble learning. Here the random subspace method is applied, as it is the usual way to create the base classifiers for the ensemble. Second, it creates lots of different subspaces. Each subspace can be directly fed into DBN to generate the high level features for SVM to create better classifier. All these classifiers could be of the diversity for the ensemble.

In the reminder of this paper, Section 2 introduces the related work. The section introduces the deep belief networks, while the new approach is presented in Section 4. The experimental results with the analysis are presented in Section 5.  Section 6 gives the conclusions and discusses the future works.

## 2. Related Work

There are lots of classifiers that can be combined to recognize the speech emotion. For example, both random forest and kernel factory are combined [23]. Both asymmetric simple partial least squares and SVM are combined [25]. The random forest, support vector machine, Naive Bayes, multilayer perception, *k*-nearest neighbors, and logistic regression are combined [26]. The neural network, decision tree, SVM, and KNN are combined [27]. Different from these methods, the classifiers to be combined can be generated from the same classification method [17]. For example, ensemble methods can be constructed through subspaces [28]. These ensemble methods do not apply DBN to learn the abstract features.

DBN has been applied to extract emotional features in speech signal automatically [29, 30] and to extract emotional features of multimodal signals (face, body gesture, voice, and physiological signals) [31]. To nicely deal with the important challenges such as distinct emotions, low quality recording, and independent affective states, DBN is combined with Fractional Calculus to extract discriminative features [32]. Besides, multitask learning is applied to leverage activation and valence information for acoustic emotion recognition using DBN framework [33]. However, in these methods, DBN are not applied within the ensemble learning framework.

The ensemble of DBN has been used for other tasks such as objects tracking [34] and facial expression recognition [11]. However, these methods do not apply random subspace and are not for speech emotion recognition. Recently, DBN have been combined to recognize the emotions from audiovisual signals [35, 36] and video [37]. However, they do not combine random subspace, DBN, and SVM for speech emotion recognition in the framework of ensemble learning.

## 3. Deep Belief Networks

**Figure 1:**
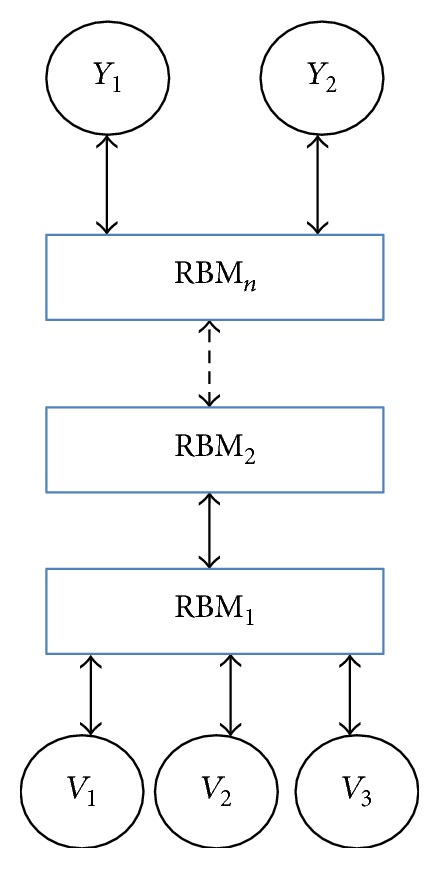
Structure of deep belief network.

DBN is composed of many RBMs in the stacking way so that it has the strong ability to learn high level representations beneficial for speech emotion recognition. It can be trained efficiently by the greedy layer-wise way. As shown as Figure 1, it begins with training the first RBM on the training data. The output of the first RBM is used as the input of the second RBM. Similarly, the third RBM is trained on the output of the second RBM. Through this way, a deep hierarchical model can be constructed that learns features from low level features to obtain the high level representation. The features extracted by DBN can serve as input to a supervised learning method such as SVM.

**Figure 2:**
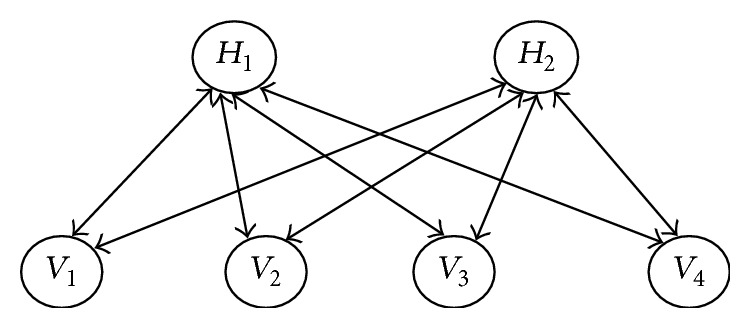
Structure of the standard RBM.

Given the training data, RBM can be trained by adjusting RBM parameters to make the probability distribution represented by RBM fit for the training data as well as possible. After successful learning, RBM provides a closed-form representation of the distribution underlying the training data. From a structural viewpoint, RBM can be regarded as a type of Markov random field that is composed of a visible and a hidden layer, shown as Figure 2, where there are links between the hidden and visible elements but links between two elements in the same layer are not permitted. The visible layer *v* represents observable data where each visible element refers to one feature of the input data. The hidden layer *h* aims to find dependencies between observed variables. *w*_*ij*_ indicates the weight between the visible unit *v*_*i*_ and the hidden unit *h*_*j*_. The joint probability distribution of (*v*, *h*) is given by the Gibbs distribution:

(1) $$\begin{array}{c} P\left(\;v,h\;\right)=\frac{e^{-E\left(v,h\right)}}{\sum_{v,h}^{}e^{-E\left(v,h\right)}}, \end{array}$$

where the energy function is defined as

(2) Ev,h=−∑i=1n∑j=1mhi∗vj∗wij+∑j=1mbj∗vj+∑i=1nci∗hi.

The involved parameters can be determined through learning from the training data using stochastic gradient ascent method. The details can be found in [38].

## 4. Random Deep Belief Networks for Ensemble

DBN is helpful to extract good speech features, but it requires the considerable skill and rich experience for human to select the optimal values for the related parameters. The tuning of these parameters is especially expensive. Besides, DBN still applies the stochastic gradient descent method to fine the parameters. This is hard to be scaled to the very deep neural network due to the “vanishing gradients” problem [9, 39]. This method does not guarantee to find the parameters that define a global minimum of the error function, but just a local minimum. It could easily be sure that there is a set of parameters that perform the best but this method cannot find out them. To deal with the issue, the ensemble learning framework is applied where the optimal parameters are not required.

**Figure 3:**
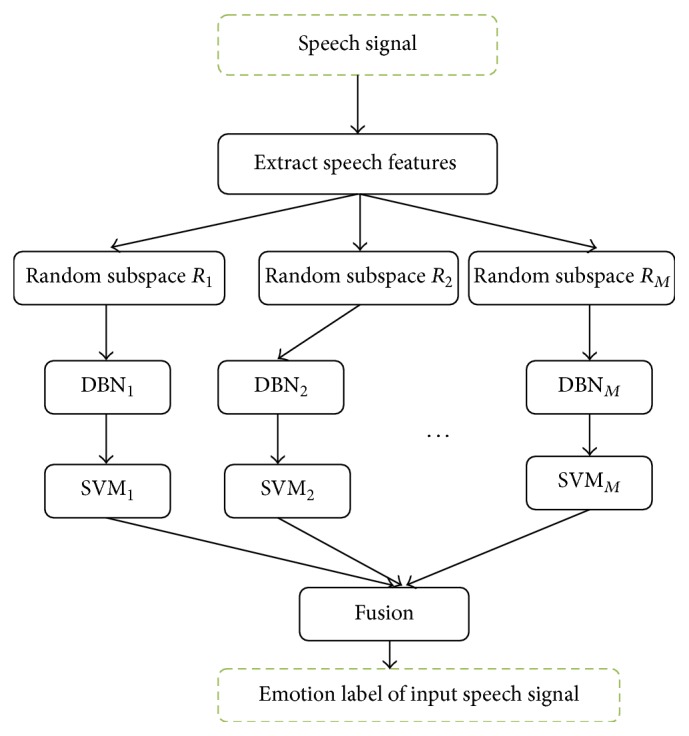
Framework of RDBN for speech emotion recognition, illustrating the method to create the base classifiers for the ensemble through random subspace, DBN, and SVM, where the majority voting is applied to perform the fusion.

Currently there are three kinds of ensemble learning applied to recognize speech emotion well. One is to train the base classifiers directly on the high dimensional feature vectors, where the base classifiers are confronted with the curse of dimensionality, leading to the fact that the ensemble classifier cannot significantly improve the effect of speech emotion recognition. To solve the problem, random subspace is applied to train the base classifiers for ensemble, where the same classification method is used. However, random subspace may not ensure providing a good description for an aspect of the speech signal and in turn affects the performance of ensemble classifier. This is because each subspace is composed of lower-level features. In such case, random subspaces need to be further proceeded by DBN. Based on the discussed factors above, this section presents an effective method for speech emotion recognition by combining random subspace, DBN, and SVM within the framework of ensemble learning. The framework of RDBN is shown as Figure 3. Its input is the speech signal and output is the emotion label of the input speech signal. RDBN first extracts the features from the input speech signals using the method discussed in the next subsection, which are then applied to create lots of random subspaces *R*_*i*_. Each *R*_*i*_ is then input DBN_*i*_ to create more abstract features for the classifier SVM_*i*_. In this way, there, *M* classifiers can be created for the ensemble. They work independently and their outputs are then fused by the majority voting. RDBN, summarized as Algorithm 1, is composed of the training stage and the testing stage. In the training stage, the speech features are extracted for all training speech signals, and then a set of base classifiers are created for the ensemble. In the testing stage, it takes the same method to extract features for the testing speech signal and then is fed up to all base classifiers. Subsequently, the majority voting is applied to make fusion, as it is simple but effective.

### 4.1. Feature Extraction

Most speech emotion recognition methods often use several approaches to extract features and then combine them, as the combined features can greatly enhance the effect of speech emotion recognition. In our approach, spectral features, prosodic features, and HuSWF (Hu Moments for Weighted Spectral Features) are combined [40]. The spectral features contain LPCC (Linear Predictor Cepstral Coefficients) [14], ZCPA (Zero Crossings with Peak Amplitudes) [41], and PLP (Perceptual Linear Predictive) [42]. Prosodic features are often used together with spectral features in speech emotion recognition, as they have good supplement effectiveness. In our approach, features of INTERSPEECH 2010 are used [43], as it contains most useful prosodic features. This feature set can be obtained by the toolbox OpenSmile [44]. HuSWF results from Hu Moments that have been widely used as the basic features [40]. It is investigated that Hu Moments have good ability to extract the differences among the emotions and can reduce the changes introduced by the sentences, the speakers, and the speaking styles.

After extracting features from a speech signal, they are transformed to a feature vector using the feature statistics methods. A larger number of global statistical functions can be used, where the used statistical functions are mean, std, max, min, kurtosis, skewness, and median, as they are the most used ones in speech emotion recognition [40]. These feature vectors are then applied to create the random subspace *R*_*i*_ as input to DBN_*i*_.

### 4.2. Base Classifiers

RDBN involves in the design of the base classifiers and the methods for combining classifiers. As SVM is extensively used for speech emotion recognition [17, 18], having advantages over GMM and HMM in the global optimality and the excellent data-dependent generalization bounds, RDBN selects it as the classification method to create the base classifiers. The diversity among the base classifiers for ensemble learning is a key issue in performance [20]. DBN is selected here to generate the variants of the input speech emotion features so as to enhance the generalization. Therefore, in our approach, random subspace, DBN, and SVM are applied to create the base classifiers.

## 5. Experiments and Validation

Experiments are conducted to validate our approach on benchmark databases that have been widely used elsewhere for SER. Some results of state-of-the-art approaches related to our approach are also compared.

### 5.1. Speech Emotion Databases

To validate RDBN, experiments are conducted on four speech databases. Berlin emotional speech database in German (EMODB) [45] is one of the most popular databases used for speech emotion recognition. This database contains 7 emotion classes. The number of each class is distributed as follows: anger (127), anxiety fear (69), boredom (81), disgust (46), happiness (71), neutral (79), and sadness (62). Surrey Audio-Visual Expressed Emotion Database (SAVEE) [46] is an English database that consists of recordings from 4 male actors in 7 different emotions. The numbers of emotion categories are distributed as anger (60), disgust (60), fear (60), happiness (60), sadness (60), surprise (60), and neutral (120). Speech Emotion Database of Institute of Automation Chinese Academy of Sciences (CASIA) [47] is a Chinese database that consists of recordings from 4 actors in 6 different emotions. The numbers of speech files for each emotion category are anger (200), fear (200), happiness (200), sadness (200), surprise (200), and neutral (200). FAU AIBO Emotion Corpus [48] consists of spontaneous recordings on German children interacting with a pet robot. The database is composed of 9959 chunks for training and 8,257 chunks for testing. It has five emotion categories. The percentage of training data from each category is as follows: anger (8.8%), emphatic (21%), neutral (56.1%), positive (6.8%), and rest (7.2%). Obviously, the distribution of the five classes is highly unbalanced.

### 5.2. Performance Evaluation Criteria

As FAU database has independent training data and testing data, they are applied directly. However, EMODB, SAVEE, and CASIA do not provide training data and testing data in advance, so that two experimental strategies are used. They are speaker-independent (SI) and speaker-dependent (SD) [42]. In SI strategy, for each fold, all utterances from one of the speakers are used for the testing data and the utterances of the remaining speakers are used as the training data. In SD strategy, all utterances of each emotion are randomly divided into five equal parts, among which four parts are taken as the training data and the remaining one is taken as the testing data. This procedure is repeated by ten times, and the average classification results across all trials were computed. The weighted accuracy (WA) and unweighted accuracy (UA) are employed to evaluate the approaches [40, 49]. WA is the total number of correctly classified testing samples of all classes averaged by the total number of testing samples. UA is the sum of all class accuracies divided by the number of classes, without considering the number of instances per class.

### 5.3. Analysis of RDBN

**Figure 4:**
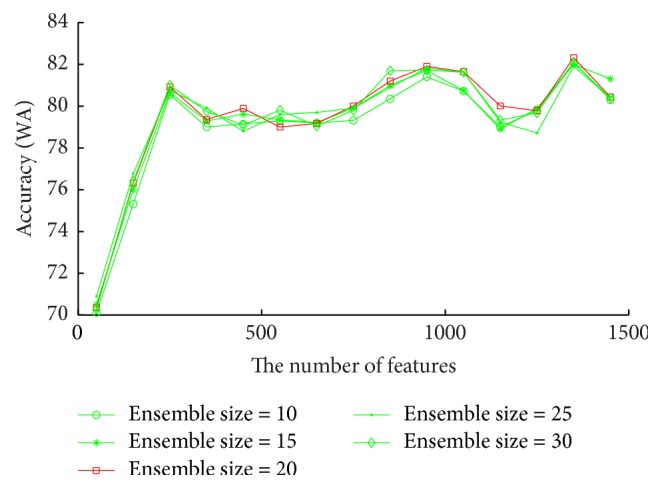
Accuracies (WA) vary with the number of features for each ensemble size on EMODB, aiming to find the optimal ensemble size and the number of features for RDBN on this database.

RDBN involves in the number of features as a parameter for each random subspace. It also depends on the ensemble size that is the number of individual classifiers for the ensemble. Generally, an ensemble method can become overtrained when the ensemble size is too large, but a smaller ensemble size always cannot reach the expected accuracy. However, the optimal values for them cannot be easily determined through theoretical analysis. They have to be tried by experiments. In experiments, the number of features varies as follows: 50, 150, 250,…, 1500. The random subspace method is applied to create 40 classifiers through training on the databases with the given number of features. After that, for each given number of features, the ensemble size varies as follows: 10, 15, 20,…, 30. The classifiers with each ensemble size are randomly selected from the previously created classifiers to build RDBN, which is then applied to perform classification. This procedure repeats ten times and then the average accuracy is computed. On the other hand, RDBN has other parameters that are also selected through experiments. In experiments, DBN takes the single layer, RBM has 80 neurons, the learning rate is 0.001, BP neural network learning rate is 0.08, the value of DropoutFraction is 0.1, and SVM with RBF kernel is applied. In experiments, SI method is applied. It can be observed from Figure 4 that when the number of features is 1350 and the ensemble size is 20, RDBN obtains the best accuracy 82.32% on EMODB database.

**Figure 5:**
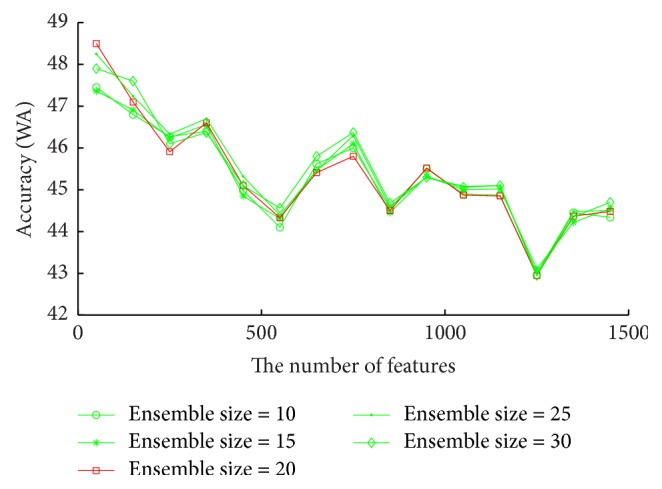
Accuracies (WA) vary with the number of features for each ensemble size on CASIA, aiming to find the optimal ensemble size and the number of features for RDBN on this database.

The results on CASIA database are shown as Figure 5. When the number of features varies from 50 to 350, RDBN have better accuracy. After that, the accuracy declines heavily along with the increase of the number of features. On this database, RDBN obtains the best accuracy 48.5% when the feature number is 50 and the ensemble size is 20.

**Figure 6:**
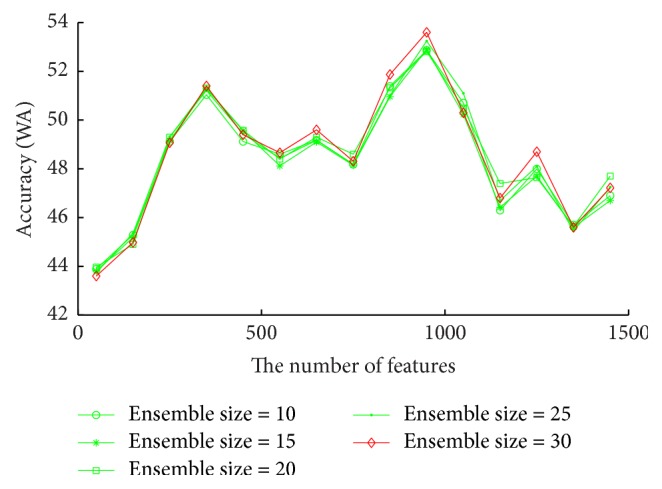
Accuracies (WA) vary with the number of features for each ensemble size on SAVEE, aiming to find the optimal ensemble size and the number of features for RDBN on this database.

It can be observed from Figure 6 on SAVEE database that when the number of features is 950 and the ensemble size is 30, RDBN obtains the best accuracy 53.6%.

In RDBN, SVM with RBF kernel (RBF-SVM) is selected to attach DBN. To validate the selection, the other classifiers are also applied to attach DBN and then make comparison through experiments, where the number of features and the ensemble sizes on each database take the same values as determined above. The compared classifiers are SVM with linear kernel (L-SVM), SOFTMAX, and KNN. It can be observed from Table 1 that RBF-SVM performs best on all databases, better than L-SVM, SOFTMAX, and KNN by 2.89%, 1.45%, and 2.22% on EMODB, respectively, by 2.08%, 2.17%, and 4% on CASIA, respectively, and by 6.6%, 2.95%, and 4.04% on SAVEE, respectively. These experimental results illustrate that it is reasonable for RDBN to choose RBF-SVM as the classifier to attach DBN.

### 5.4. Performance of RDBN on EMODB, CASIA, and SAVEE

In order to further analyze the generalization ability of RDBN, the confusion matrixes of experimental results on EMODB, CASIA, and SAVEE are obtained by the averaging ten experimental results, where seven emotions are considered: anger, disgust, fear, happy, sadness, surprise, and neutral. In confusion matrix, the row means the true emotion classes while the column indicates the predicted emotion classes. It can be observed from Table 2 that, on EMODB, RDBN performs best on the sadness emotion with accuracy up to 96.16% and performs well on angry, indicating that the negative emotion can be recognized nicely by RDBN. To our surprise, the happy emotion cannot be nicely recognized whose accuracy is only 59.17%. From Table 3, it can be concluded on CASIA that our method performs recognition well on angry and sad emotions. Particularly, its performance on angry emotion reaches up to 72.5%. On the other hand, it has poor effects on the recognition of both fear and surprise emotions with accuracy down to 33%. On SAVEE, it can be observed from Table 4 that RDBN performs better on the happy, neutral, and surprise emotion. It is surprising that recognition of the neutral emotion achieves accuracy of 74.08%, while the effect on angry and sadness is poor with the accuracy about 44%. These experimental results indicate that on the whole RDBN can be applied to recognize the negative emotions. But the conclusion is not consistent on all three databases. This is because the samples distributions of different emotions on all three databases are not the same.

### 5.5. Compared Methods on EMODB, CASIA, and SAVEE

To further validate RDBN, many methods are compared on the speech emotion databases. They are the original DBN with one layer (SLDBN), DBN with two layers (DLDBN), and DBN with three layers (TLDBN). Both KNN and SVM are also compared, as they are often applied on speech emotion recognition [3, 22]. Additionally, the best base classifier of RDBN, denoted as BASE, is also compared. All classifiers based on DBN have the same parameters as that of RDBN. In the experiments, SI is used. All other parameters are determined through tenfold cross-validation. The experimental results are shown in Table 5. It can be concluded that RDBN obviously outperforms the other classifiers on all databases. It has the accuracies on EMODB, CASIA, and SAVEE higher than BASE by 2.71%, 12.33%, and 9.02%, respectively, indicating that the ensemble learning is effective. On the other hand, SLDBN significantly outperforms DLDBN and TLDBN on three databases.

For example, it is better than DLDBN by 9.48%, 10%, and 23.18% on EMODB, CASIA, and SAVEE, respectively. The reason is that the layers of deep belief network depend on the size of the training database whereas used databases are smaller, not enough to train the classifier well. Finally, RDBN obtains the accuracies higher than L-SVM by 1.13%, 6.42%, and 7.35%, respectively, on three databases. It is also higher than KNN by 11.58%, 14.17%, and 10.47%, respectively, on three databases.

### 5.6. Performance of RDBN on FAU

FAU differs from EMODB, CASIA, and SAVEE in that it has different speech emotion labels such as anger, stress, positive, neutral, and others. Secondly, FAU is constructed by two school children. The speech signal data from one school is taken as the training database, while the other is taken as the testing database. Both databases are distributed unevenly, requiring that the training database must be balanced such as by downsampling method [4].

**Figure 7:**
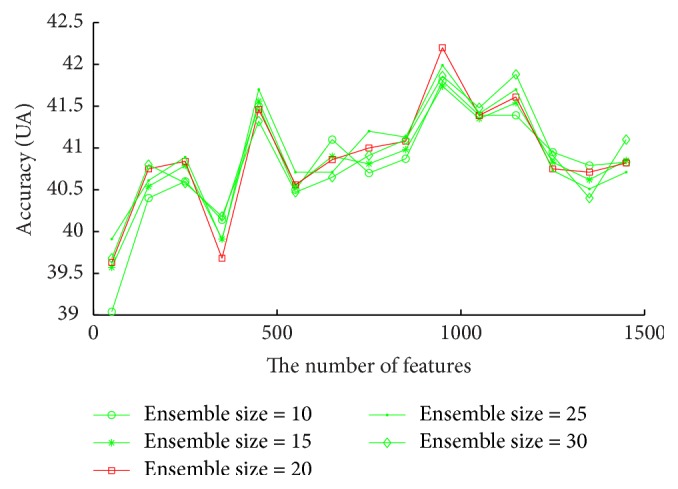
Accuracies (WA) vary with the number of features for each ensemble size on FAU database, aiming to find the optimal ensemble size and the number of features for RDBN on this database.

Downsampling reduces the size of the majority class to the size of the minority class. As the testing database is unbalanced, if WA is still taken as the performance criteria, the classifier performs well on the class types with the large number of samples and bad on the ones with the small number of samples, leading to the good recognition results in terms of WA. However, this conclusion would be biased. Hence instead of WA, UA is applied to evaluate RDBN, where its optimal parameters are determined in advance through experiments. It can be concluded from Figure 7 that when the number of features is 950 and the ensemble size is 20, RDBN obtains the best recognition accuracy with 42.2%. Using optimal parameters, all methods are compared on FAU database. The experimental results are shown in Table 6. It can be concluded that RDBN performs best among all methods, better than the second one SLDBN by 1.68%.

Furthermore the accuracy decreases along with the number of layers from single to three, illustrating that the database has not enough samples. Secondly, RDBN outperforms BASE by 3.1%, illustrating that the ensemble learning is definitely superior to its single classifier. Finally, RDBN has certain advantages in speech emotion recognition over the classical methods, higher than L-SVM by 4.83% and KNN by 6.5%.

It can be concluded from the above experimental results that RDBN consistently outperforms DBN, SVM, and KNN for speech emotion recognition. It is also seen that all approaches do not obtain the much nice performance on the databases. The main reason is that the training database for our approach is not large enough to contain all kinds of samples, as there is a strong demand for more labelled speech signals in order to better understand human emotions and the way they are expressed. Unfortunately, emotion databases are typically small due to the manual process of annotating them with emotional labels. These problems can be solved using semisupervised learning methods in the future work [50].

## 6. Conclusion and Future Work

This paper presents a random deep belief network (RDBN) ensemble method for speech emotion recognition. It has the following advantages. Firstly, it has the ability to overcome the curse of dimensionality problem due to random subspace used. Secondly, it has the potential ability to obtain better performance when the larger training databases can be available, as it applies the deep belief network on random subspaces. Thirdly, it takes SVM as the base classifier which can output the probability of a testing sample belonging to each emotion instead of the concrete emotion label. This makes RDBN able to better deal with the uncertainty information in the fusion of the base classifiers. Finally, RDBN is based on the ensemble learning so that it can perform the complicated recognition tasks. However, our approach is still challenged by the lower accuracies on the speech emotion databases. This possibly results from the smaller training databases and the poor diversity. In the future, the larger speech emotion database will be constructed to train RDBN, as RDBN can be nicely scaled to the larger data with the better performance. On the other hand, the diversity of the ensemble has not been considered here, which will be emphasized to further enhance the performance of our approach.

## Figures and Tables

**Algorithm 1:**
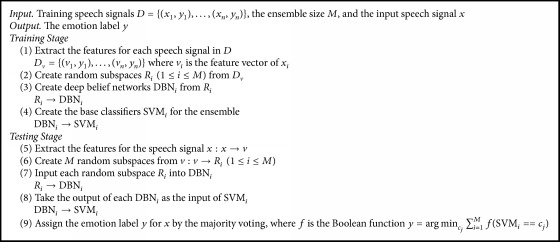
RDBN.

**Table 1:** Accuracies of different classifiers attached to DBN.

	RBF-SVM	L-SVM	SOFTMAX	KNN
EMODB	82.32	79.43	80.87	80.10
CASIA	48.50	46.42	46.33	44.50
SAVEE	53.60	47.00	50.65	49.46

**Table 2:** Confusion matrix of RDBN on EMODB, illustrating the ability of RDBN on each emotion class.

Emotion	Happy	Neutral	Angry	Sadness	Fear	Surprise	Disgust
Happy	59.17	0.00	28.15	0.00	3.66	0.00	9.01
Neutral	1.27	92.14	0.00	0.00	1.27	5.33	0.00
Angry	8.13	0.00	88.64	0.00	0.87	0.00	2.36
Sadness	0.00	0.32	0.00	96.16	0.00	3.52	0.00
Fear	8.70	4.20	4.80	1.45	75.06	0.00	5.80
Surprise	0.62	11.10	0.00	6.17	0.86	79.40	1.85
Disgust	15.87	2.17	2.17	2.17	2.39	2.17	73.05

**Table 3:** Confusion matrix of RDBN on CASIA, illustrating the ability of RDBN on each emotion class.

Emotion	Angry	Fear	Happy	Neutral	Sadness	Surprise
Angry	72.50	2.00	12.00	2.50	0.50	10.50
Fear	5.00	33.00	3.50	6.50	42.50	9.50
Happy	11.00	3.50	57.50	6.50	13.00	8.50
Neutral	4.50	9.50	32.50	35.50	12.50	5.50
Sadness	0.50	24.00	5.00	6.00	59.50	5.00
Surprise	17.50	18.00	17.00	6.50	8.00	33.00

**Table 4:** Confusion matrix of RDBN on SAVEE, illustrating the ability of RDBN on each emotion class.

Emotion	Angry	Disgust	Fear	Happy	Neutral	Sadness	Surprise
Angry	44.00	26.67	4.67	18.33	4.67	0.00	1.67
Disgust	8.00	44.17	1.67	0.00	30.50	7.33	8.33
Fear	2.33	11.17	31.17	20.17	13.50	2.67	19.00
Happy	9.67	6.33	14.00	59.00	3.33	0.00	7.67
Neutral	0.00	24.42	0.42	0.00	74.08	1.08	0.00
Sadness	3.33	24.00	0.00	0.00	28.33	44.33	0.00
Surprise	0.00	8.67	11.50	14.33	9.83	1.67	54.00

**Table 5:** Accuracies (WA) of the compared methods on three databases, illustrating the superiority of RDBN to the other methods.

	L-SVM	KNN	SLDBN	DLDBN	TLDBN	RDBN	BASE
EMODB	81.19	70.74	72.84	53.85	24.59	82.32	79.61
CASIA	42.08	34.33	38.50	29.50	18.25	48.50	36.17
SAVEE	46.25	43.13	30.42	20.62	25.00	53.60	44.58

**Table 6:** Accuracies (UA%) of the compared methods on FAU database, illustrating the superiority of RDBN to the other methods.

	L-SVM	KNN	SLDBN	DLDBN	TLDBN	RDBN	BASE
FAU	37.37	35.70	40.52	30.50	30.10	42.20	39.10
